# Evolution and the Children of Freedom

**Published:** 1908-04

**Authors:** 


### EDITORIALS

The Business Office of the JOURNAL-RECORD is 1 1-2 to 5 1-2 South Broad St.
The Editorial Office is 1014-15 Century Bldg.
Address all Business Communications to Journal-Record of Medicine, 1 1-2 to 5 1-2 South Broad Street.
Make remittances payable to THE JOURNAL-RECORD OF MEDICINE.
On matters pertaining to the Editorial and Original communications, address Edgar G. Ballenger, M. D., Atlanta, Ga.
Reprints of original articles will be furnished at cost price. Requests for the same should always be made in THE MANUSCRIPT.
We will present, postpaid, on request, to each contributor of an original article, twenty (20) marked copies of THE JOURNAL-RECORD OF MEDICINE containing such article.

### EVOLUTION AND THE CHILDREN OF FREEDOM.

The present writer recently had the pleasure of taking part in a debate upon "Freedom and the Republic," in which he ventured to assert that the nation's children, being its most precious possession, should receive at least a modicum of the national care. He thought the community should stand behind the parent, and the nation behind the community; and that where the parent failed in his duty to the child the community and the nation should be prepared to deal with both the parent and the child according to their deserts. What the deserts of the child may be was the point at which the present writer and the accomplished essayist of the meeting crossed words. It was argued that the child had no rights beyond those provided by the law of Evolution, which, with red ravening maw ruthlessly cuts down all who might be unfit to wage successfully an individual war against that nature which is "so careful of the type, so careless of the single life."

This appears to be but a sad commentary upon the kindly religions, the hospitals, the homes for the friendless, the orphanages, and the thousand charities which grace the land. Is it well that the child, absolutely irresponsible for his position or even for his existence, should suffer cold and hunger, ignorance and disease, and grow up but the shadow of the man he might have been because evolution has led mankind through dark and devious paths sometime, and still perforce must tread therein? or was the gentleman laboring under a misunderstanding in relation to the Law of Evolution? It has doubtless been true that the battle is to the strong, and that the fittest have the greatest chance of survival, and always must be true. But there are various forms of strength. The wounded buffalo calf separated from the herd was the sure prey of the relentless wolf; the strength of the antelope lies in its power to run away; some richly hued insects escape because they resemble the stinging wasp. The buffalo teaches us the value of combination; the antelope of removing our kind from danger, and the mimetic insect that there are other ways of making strong the weakling besides the heathen method of exposure. The Law of Evolution has already taken on many forms, and it is growing now in its most beautiful and bountiful shape. It ceased some centuries ago to be of necessity a cruel law. Man long ago began to discover that he may one day better evolve through kindliness than through capacity; through combination than through rivalry; thrift than through extravagance. It lies in great part with us doctors to guie this sentiment aright, and to see to it that the stream of less robust humanity, be neither in quantity nor quality a danger to the whole. This is to be accomplished by such a wise and far-seeing use of the art of preventive medicine in its widest sense that fewer weaklings shall be born, and these shall be so carefully tended and matured that our cities shall harbor no more of those thousands of degenerates who degrade race, are a source of constant menace and expense, and a standing rebuke to our civilization.

